# Supplementary material for: Genetic Diversity of Durum Wheat (Triticum turgidum L. ssp. durum, Desf) Germplasm as Revealed by Morphological and SSR Markers
Source: Genes (Basel). 2023 May 26;14(6):1155. doi: 10.3390/genes14061155 (PMC10298645; doi:10.3390/genes14061155)
Supplement: Supplementary file 1 [file genes-14-01155-s001.zip › genes-2409555-supplementary.pdf]

genes
MDPI

Supplementary Files

Genetic Diversity of Durum Wheat (*Triticum turgidum* L. ssp. *durum*, Desf) Germplasm as Revealed by Morphological and SSR Markers

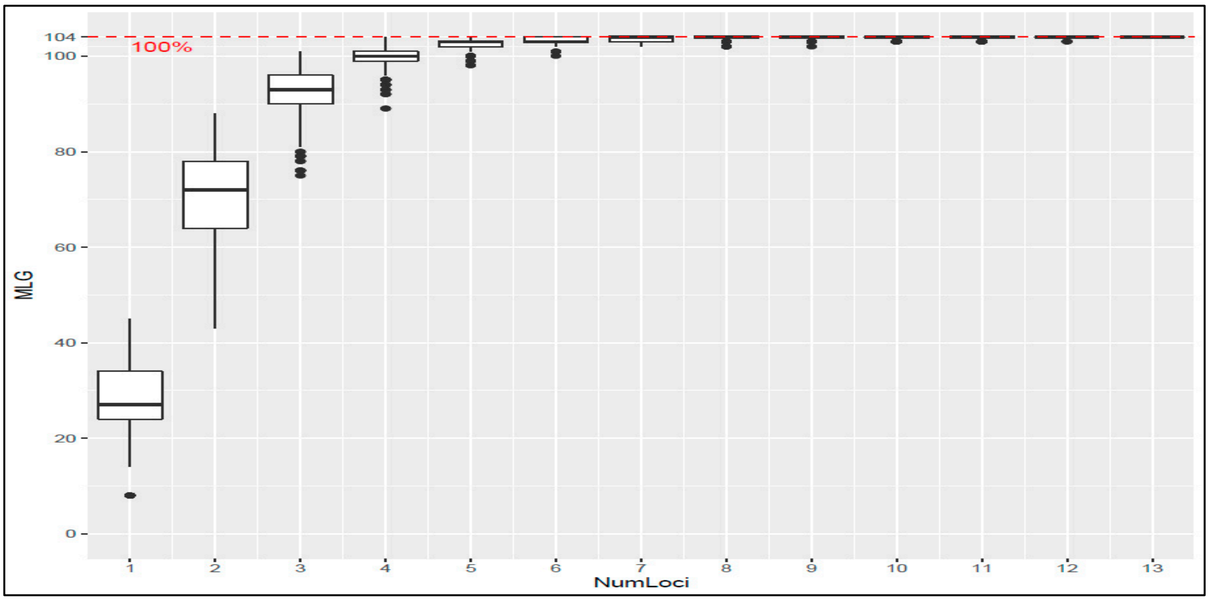

NumLoci is number of loci; MLG is Multiple Loci Genotypes.

Figure S1. The genotype accumulation curve of fourteen simple sequence repeat (SSR) markers across 104 Ethiopian durum wheat genotypes.

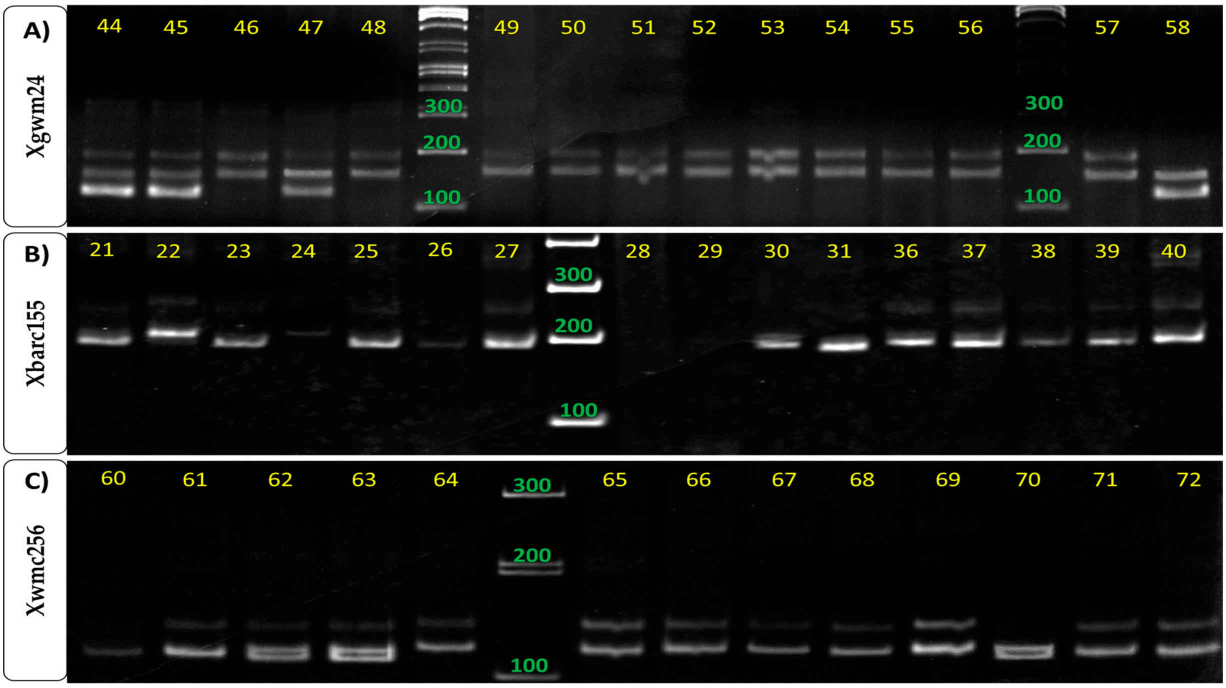

Figure S2. Electrophoresed gel image of three simple sequence repeat (SSR) markers: Xwmc24 (A); Xbarc155 (B); and Xwmc256 (C).

genes

Table S1. Description of 104 Ethiopian durum wheat genotypes used in the molecular diversity study.

Genotype code	Accession name	Population	Region	Altitude	Latitude	Longitude
1	5617	West Gojam	Amhara	2001—2400	10.57	37.48
2	5198	West Gojam	Amhara	2001—2400	10.57	37.48
3	5202	West Gojam	Amhara	2001—2400	10.57	37.48
4	5340	West Gojam	Amhara	2001—2400	10.57	37.48
5	5207	West Gojam	Amhara	2001—2400	10.57	37.48
6	5397	West Gojam	Amhara	2001—2400	10.57	37.48
7	5204	West Gojam	Amhara	2001—2400	10.57	37.48
8	5291	West Gojam	Amhara	2001—2400	10.57	37.48
9	6974	East Gojam	Amhara	2401—2800	10.62	38.18
10	7801	East Gojam	Amhara	2001—2400	11.08	37.87
11	7823	East Gojam	Amhara	2401—2800	10.3	38.2
12	7826	East Gojam	Amhara	2401—2800	10.3	38.2
13	7798	East Gojam	Amhara	2401—2800	10.5	38.17
14	7822	East Gojam	Amhara	2401—2800	10.3	38.2
15	7828	East Gojam	Amhara	2401—2800	10.3	38.2
16	6975	East Gojam	Amhara	2401—2800	10.58	38.17
17	5294	North Gondar	Amhara	2401-2800	12.72	37.5
18	5213	North Gondar	Amhara	2001—2400	12.63	37.47
19	5214	North Gondar	Amhara	2001—2400	12.63	37.47
20	5470	North Gondar	Amhara	> 2800	12.92	37.75
21	5342	North Gondar	Amhara	2401—2800	12.72	37.5
22	5219	North Gondar	Amhara	2001—2400	12.63	37.47
23	5125	North Gondar	Amhara	> 2800	12.92	37.78
24	5342	North Gondar	Amhara	2401—2800	12.72	37.5
25	7532	South Wello	Amhara	2001—2400	11.35	39.3
26	7568	South Wello	Amhara	2401—2800	10.78	39.45
27	243698	South Wello	Amhara	> 2800	11.03	39.25
28	7378	South Wello	Amhara	2001—2400	11.05	39.75
29	7581	South Wello	Amhara	2401—2800	10.42	39.28
30	7580	South Wello	Amhara	2401—2800	10.62	39.58
31	7375	South Wello	Amhara	< 2000	11.1	39.75
32	5597	North Shewa	Amhara	2001—2400	8.83	39.32
33	5168	North Shewa	Amhara	2001—2400	8.83	39.32
34	5892	North Shewa	Amhara	2401—2800	9.8	39.2
35	5047	North Shewa	Oromia	> 2800	9.77	38.77
36	7104	North Shewa	Oromia	> 2800	9.32	39.27
37	5309	North Shewa	Oromia	2401—2800	9.05	39.07
38	5609	North Shewa	Oromia	2401—2800	9.05	39.07
39	5251	North Shewa	Oromia	2401—2800	9.05	39.07
40	5600	North Shewa	Oromia	> 2800	9.78	38.7
41	5568	East Shewa	Oromia	2001—2400	8.88	38.82
42	5432	East Shewa	Oromia	2001—2400	8.78	39.25

43	242791	East Shewa	Oromia	2001—2400	9	39.12
44	5574	East Shewa	Oromia	2401—2800	9.02	39.05
45	5623	East Shewa	Oromia	2001—2400	8.85	38.87
46	214370	East Shewa	Oromia	< 2000	8.83	38.95
47	5548	East Shewa	Oromia	2001—2400	8.9	39.02
48	5502	East Shewa	Oromia	2001—2400	9.02	39.05
49	5567	East Shewa	Oromia	2001—2400	8.85	39.07
50	5420	East Shewa	Oromia	> 2800	9.02	39.25
51	5354	West Shewa	Oromia	2001—2400	8.85	38.5
52	7210	West Shewa	Oromia	2401—2800	8.97	38.03
53	5537	West Shewa	Oromia	2401—2800	8.53	37.97
54	5149	West Shewa	Oromia	2001—2400	8.85	38.5
55	5554	West Shewa	Oromia	2001—2400	8.85	38.5
56	5043	West Shewa	Oromia	< 2000	8.83	38.37
57	226897	West Shewa	Oromia	2001—2400	8.95	37.87
58	5025	West Shewa	Oromia	2001—2400	8.97	37.77
59	5098	East Harerge	Oromia	2001—2400	9.43	41.8
60	5278	East Harerge	Oromia	2001—2400	9.27	41.8
61	5103	East Harerge	Oromia	2001—2400	9.43	41.8
62	5018	East Harerge	Oromia	2001—2400	9.43	41.8
63	5119	East Harerge	Oromia	2001—2400	9.45	41.9
64	5095	East Harerge	Oromia	2001—2400	9.42	41.68
65	5104	East Harerge	Oromia	< 2000	9.58	42.12
66	5109	East Harerge	Oromia	< 2000	9.77	41.8
67	8072	Bale	Oromia	< 2000	7.13	40.72
68	238891	Bale	Oromia	< 2000	7	39.97
69	239693	Bale	Oromia	2001—2400	7.03	40.63
70	5006	Bale	Oromia	< 2000	7	39.97
71	5146	Bale	Oromia	2401—2800	7.33	39.75
72	5011	Bale	Oromia	> 2800	7	39.12
73	5017	Bale	Oromia	> 2800	7	39.83
74	7031	Arsi	Oromia	> 2800	8.25	39.78
75	7014	Arsi	Oromia	2401—2800	7.7	40.13
76	222393	Arsi	Oromia	2401—2800	7.78	39.65
77	7046	Arsi	Oromia	2401—2800	8.08	39.63
78	7317	Arsi	Oromia	2401—2800	7.13	39.3
79	7069	Arsi	Oromia	2401—2800	7.67	39.87
80	5163	Arsi	Oromia	2401—2800	7.82	39.78
81	3540	Central Tigray	Tigray	< 2000	14.12	38.48
82	5453	Central Tigray	Tigray	2001—2400	14.12	38.48
83	5669	Central Tigray	Tigray	2001—2400	14.17	38.7
84	243717	Central Tigray	Tigray	2401—2800	13.63	39.12
85	5653	Central Tigray	Tigray	2401—2800	14.12	38.48
86	5468	Central Tigray	Tigray	< 2000	14.15	38.92
87	2211	Central Tigray	Tigray	2001—2400	14.12	38.48

88	5348	Southern Tigray	Tigray	2401—2800	13.45	39.55
89	5136	Southern Tigray	Tigray	2001—2400	13.28	39.52
90	3540	Southern Tigray	Tigray	2001—2400	13.5	39.55
91	5573	Southern Tigray	Tigray	2001—2400	13.5	39.55
92	5286	Southern Tigray	Tigray	2001—2400	13.75	38.6
93	5394	Southern Tigray	Tigray	> 2800	12.97	39.52
94	5526	Southern Tigray	Tigray	2401—2800	13.5	39.55
95	Werer	Cultivars	Cultivars			
96	Robe	Cultivars	Cultivars			
97	Ejersa	Cultivars	Cultivars			
98	Tate	Cultivars	Cultivars			
99	Arandato	Cultivars	Cultivars			
100	Yerer	Cultivars	Cultivars			
101	Ilani	Cultivars	Cultivars			
102	Boohai	Cultivars	Cultivars			
103	Kilinto	Cultivars	Cultivars			
104	Tob-66	Cultivars	Cultivars			

Table S2. The frequency distribution of phenotypic classes of ten morphological traits in 104 Ethiopian durum wheat genotypes by entire collection, populations, regions and altitudinal classes.

Trait	Phenotypic class	Collection	Populations													Regions			Altitudinal classes			
			West Gojam	East Gojam	North Gondar	South Wello	North Shewa	East Shewa	West Shewa	East Harerge	Bale	Arsi	Central Tigray	Southern Tigray	Cultivars	Amhara	Oromia	Tigray	< 2001	2001 – 2401	2401 – 2801	> 2801
DH	<65	21.6	25.0	6.3	15.6	21.4	22.2	5.0	12.5	28.1	21.4	21.4	3.6	21.4	67.5	16.9	17.9	12.5	12.5	19.6	13.7	18.2
	66-74	44.5	53.1	50.0	46.9	39.3	55.6	47.5	46.9	43.8	39.3	28.6	53.6	42.9	30.0	47.8	44.0	48.2	40.0	47.6	46.0	45.5
	>75	33.9	21.9	43.8	37.5	39.3	22.2	47.5	40.6	28.1	39.3	50.0	42.9	35.7	2.5	35.3	38.0	39.3	47.5	32.7	40.3	36.4
DM	<120	0.0	0.0	0.0	0.0	0.0	0.0	0.0	0.0	0.0	0.0	0.0	0.0	0.0	0.0	0.0	0.0	0.0	0.0	0.0	0.0	0.0
	121-135	17.3	18.8	21.9	9.4	7.1	16.7	10.0	6.3	31.3	28.6	10.7	14.3	25.0	25.0	14.7	16.8	19.6	22.5	15.5	14.5	20.5
	>136	82.7	81.3	78.1	90.6	92.9	83.3	90.0	93.8	68.8	71.4	89.3	85.7	75.0	75.0	85.3	83.2	80.4	77.5	84.5	85.5	79.5
SPS	<17	13.2	9.4	9.4	9.4	7.1	11.1	17.5	12.5	12.5	10.7	32.1	7.1	21.4	12.5	8.8	16.3	14.3	22.5	11.3	13.7	11.4
	17-21	60.3	71.9	53.1	62.5	75.0	50.0	55.0	46.9	68.8	60.7	42.9	60.7	53.6	80.0	63.2	54.9	57.1	50.0	63.7	54.8	54.5
	>21	26.4	18.8	37.5	28.1	17.9	38.9	27.5	40.6	18.8	28.6	25.0	32.1	25.0	7.5	27.9	28.8	28.6	27.5	25.0	31.5	34.1
PLH	<85	3.1	0.0	0.0	0.0	7.1	5.6	0.0	0.0	0.0	0.0	0.0	0.0	0.0	22.5	1.5	1.1	0.0	0.0	0.6	1.6	2.3
	85-105	36.5	28.1	40.6	46.9	35.7	41.7	37.5	25.0	40.6	39.3	21.4	39.3	50.0	30.0	38.2	34.2	44.6	32.5	36.9	33.9	52.3
	106-124	49.0	62.5	56.3	28.1	46.4	41.7	55.0	68.8	46.9	42.9	46.4	50.0	46.4	45.0	49.3	50.0	48.2	47.5	54.8	51.6	25.0
	>125	11.3	9.4	3.1	25.0	10.7	11.1	7.5	6.3	12.5	17.9	32.1	10.7	3.6	2.5	11.0	14.7	7.1	20.0	7.7	12.9	20.5
TKW	<37	15.1	12.5	40.6	18.8	10.7	8.3	25.0	9.4	12.5	0.0	17.9	21.4	14.3	5.0	19.9	13.0	17.9	5.0	17.3	16.1	22.7
	37-44.9	51.4	65.6	46.9	65.6	64.3	63.9	52.5	68.8	31.3	50.0	46.4	50.0	67.9	7.5	61.8	51.1	58.9	32.5	60.7	58.1	54.5
	45-53	26.4	21.9	12.5	15.6	25.0	22.2	20.0	15.6	31.3	46.4	32.1	28.6	17.9	52.5	18.4	27.7	23.2	50.0	18.5	22.6	22.7
	>53	7.0	0.0	0.0	0.0	0.0	5.6	2.5	6.3	25.0	3.6	3.6	0.0	0.0	35.0	0.0	8.2	0.0	12.5	3.6	3.2	0.0
GY	<6	12.7	18.8	9.4	9.4	17.9	13.9	22.5	21.9	3.1	0.0	32.1	3.6	7.1	5.0	14.0	15.8	5.4	20.0	13.1	12.9	11.4
	6--9	52.4	46.9	53.1	59.4	50.0	52.8	52.5	53.1	53.1	71.4	39.3	46.4	67.9	40.0	52.9	53.3	57.1	60.0	54.2	50.0	56.8
	9--12	30.3	34.4	37.5	25.0	21.4	25.0	25.0	18.8	43.8	28.6	28.6	39.3	25.0	40.0	28.7	28.8	32.1	20.0	29.2	32.3	29.5
	>12	4.6	0.0	0.0	6.3	10.7	8.3	0.0	6.3	0.0	0.0	0.0	10.7	0.0	15.0	4.4	2.2	5.4	0.0	3.6	4.8	2.3

genes
MDPI

GC	<21	9.4	9.4	15.6	9.4	7.1	2.8	5.0	18.8	9.4	17.9	0.0	10.7	0.0	15.0	9.6	9.2	5.4	7.5	10.1	7.3	9.1
	21-27.9	44.7	53.1	43.8	46.9	60.7	38.9	25.0	37.5	50.0	32.1	25.0	50.0	64.3	57.5	51.5	33.2	57.1	47.5	41.7	47.6	34.1
	28-35	38.0	37.5	34.4	43.8	28.6	47.2	50.0	40.6	28.1	32.1	53.6	39.3	28.6	27.5	36.0	42.9	33.9	30.0	41.1	36.3	47.7
	>35	7.9	0.0	6.3	0.0	3.6	11.1	20.0	3.1	12.5	17.9	21.4	0.0	7.1	0.0	2.9	14.7	3.6	15.0	7.1	8.9	9.1
GPC	<13	43.8	43.8	34.4	46.9	64.3	44.4	27.5	46.9	53.1	42.9	17.9	46.4	50.0	52.5	47.1	38.0	48.2	50.0	42.9	41.1	40.9
	13-16	46.2	53.1	59.4	46.9	28.6	41.7	52.5	46.9	31.3	39.3	64.3	46.4	39.3	47.5	47.1	46.2	42.9	35.0	45.8	51.6	40.9
	>16	10.1	3.1	6.3	6.3	7.1	13.9	20.0	6.3	15.6	17.9	17.9	7.1	10.7	0.0	5.9	15.8	8.9	15.0	11.3	7.3	18.2
SPD	Lax	27.4	28.1	28.1	31.3	32.1	27.8	42.5	21.9	34.4	17.9	57.1	21.4	17.9	0.0	28.7	34.8	19.6	32.5	23.8	39.5	27.3
	Intermediate	51.9	50.0	71.9	62.5	46.4	61.1	50.0	43.8	62.5	67.9	32.1	64.3	67.9	7.5	60.3	51.1	66.1	57.5	61.3	50.8	54.5
	Dense	20.7	21.9	0.0	6.3	21.4	11.1	7.5	34.4	3.1	14.3	10.7	14.3	14.3	92.5	11.0	14.1	14.3	10.0	14.9	9.7	18.2
VTR	Non- vitreous	4.8	18.8	6.3	0.0	0.0	0.0	0.0	12.5	6.3	0.0	0.0	0.0	7.1	10.0	5.9	3.3	3.6	0.0	8.3	1.6	0.0
	Partly- vitreous	14.4	6.3	12.5	31.3	0.0	0.0	15.0	12.5	12.5	28.6	0.0	14.3	35.7	20.0	11.8	12.0	25.0	15.0	15.5	9.7	18.2
	Vitreous	80.8	75.0	81.3	68.8	100	100	85.0	75.0	81.3	71.4	100	85.7	57.1	70.0	82.4	84.8	71.4	85.0	76.2	88.7	81.8
